# *Rugitermes tinto*: A new termite (Isoptera, Kalotermitidae) from the Andean region of Colombia

**DOI:** 10.3897/zookeys.963.55843

**Published:** 2020-08-24

**Authors:** Rudolf H. Scheffrahn, Olga Patricia Pinzón Florian

**Affiliations:** 1 Fort Lauderdale Research and Education Center, University of Florida, 3205 College Avenue Davie, Florida 33314, USA University of Florida Davie United States of America; 2 Universidad Distrital “Francisco José de Caldas,” Cra. 5E 3 15-82 Bogotá, Distrito Especial, Colombia Universidad Distrital “Francisco José de Caldas” Bogotá Colombia

**Keywords:** frontolateral ridges, imago, new species, soldier, South America, taxonomy

## Abstract

The imago and soldier castes of a new *Rugitermes* Holmgren, 1911 species, *R. tinto***sp. nov.** are described. It is the ninth species of *Rugitermes* from South America and the first record of this genus from Colombia. Unlike its congeners, the soldier of *R. tinto* has very dark head capsule pigmentation and acute protuberances projecting from frontolateral ridges.

## Introduction

**Figure 1.**
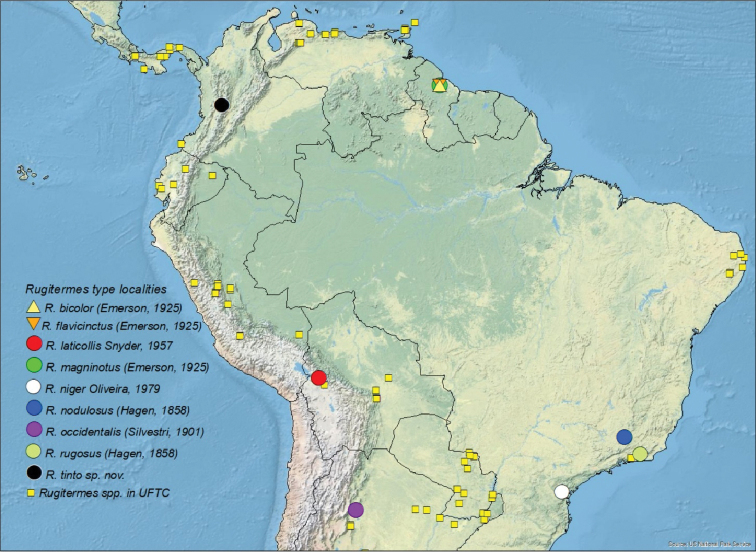
Type localities for all *Rugitermes* species described from South America and collection localities for *Rugitermes* spp. in the University of Florida Termite Collection (UFTC).

*Rugitermes* Holmgren, 1911 is a widespread termite genus in Central and South America (Scheffrahn 2019a) where twelve species are described (Krishna et al. 2013). A curious thirteenth species, *R. athertoni* (Light, 1932), occurs in Oceania. Eight species of *Rugitermes* are currently known from South America: *R. bicolor* (Emerson, 1925) from Amazonia (Scheffrahn 2019b), *R. laticollis* Snyder, 1957 from the Andean highlands (Scheffrahn 2015), *R. flavicinctus* (Emerson, 1925) and *R. magninotus* (Emerson, 1925) from Guyana, *R. niger* Oliveira, 1979, *R. nodulosus* (Hagen, 1858), and *R. rugosus* (Hagen, 1858) from southern Brazil, and *R. occidentalis* (Silvestri, 1901) from Argentina. The type localities for the South American species, including *Rugitemes tinto* sp. nov., are given in Fig. 1.

In South America, only four kalotermitid genera have soldiers with virtually no head capsule phragmosis: *Comatermes*, *Incisitermes*, *Neotermes*, and *Rugitermes* (Scheffrahn, 2019a). In his generic description, Krishna (1961) wrote that the *Rugitermes* soldier can be differentiated from the other three genera by the former’s “prominent ridge…present in front of and medial to the antennal socket”. The *Rugitermes* imago is distinguished by its “short, sclerotized median vein in the forewing which joins the radial sector very close to the wing suture” (Krishna 1961). The coloration of the head and pronotum contrast sharply in the majority of species.

Herein, we describe the imago and soldier of *Rugitermes tinto* sp. nov. This is the first record of *Rugitermes* from Colombia.

## Material and methods

Microphotographs were taken as multi-layer montages using a Leica M205C stereomicroscope controlled by Leica Application Suite version 3 software. Preserved specimens were taken from 85 % ethanol and suspended in a pool of Purell Hand Sanitizer to position the specimens on a transparent Petri dish background.

## Taxonomy

### Rugitermes tinto

Taxon classificationAnimaliaBlattodeaKalotermitidae

Scheffrahn & Pinzón Florian sp. nov.

78DF2D45-8FF9-5E13-8138-5C30978B59AA

http://zoobank.org/601947D7-EF34-4209-A9A2-7B43A11BF317

Figures 2 , 3

#### Diagnosis.

The soldier of *R. tinto* is the only non-phragmotic kalotermitid worldwide with dark coloration of the anterior head capsule. Aside from head color, it differs from congeneric soldiers in that the anterolateral corners of the frontal ridges project to form acute angles. In other South American (Fig. 4), Central American, and Oceanian *Rugitermes*, the anterolateral corners of the frontal ridges are either at right or obtuse angles.

Among South American species, the imago of *R. tinto*, with contrasting coloration of the head and pronotum, is similar to *R. bicolor*, *R. flavicinctus*, *R. magninotus*, and *R. nodulosus*. Of these, the imago of *R. bicolor* is larger, while that of *R. flavicinctus* is smaller than *R. tinto* and the head/pronotum coloration of the former two are similar to *R. magninotus*. The imago of *R. magninotus* has a brown head and a yellow pronotum while the imago of *R. tinto* has a black head and a brownish pronotum. The distribution of *R. nodulosus* is known only from southern Brazil (Minas Gerais).

#### Description.

**Figure 2.**
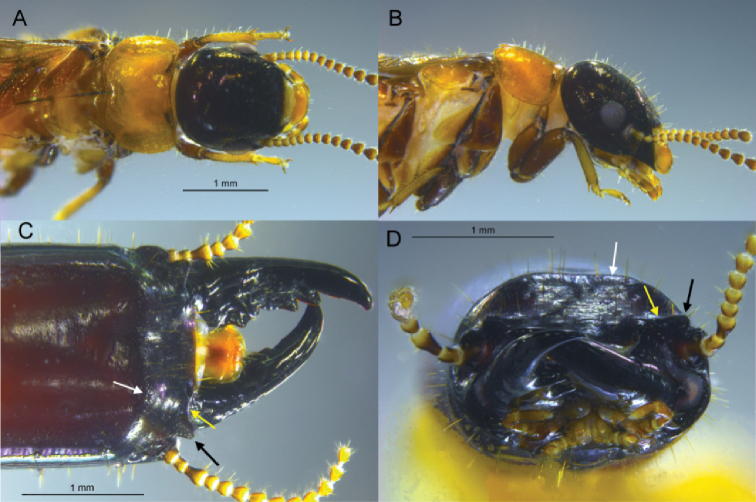
*Rugitermes tinto* sp. nov. **A** Dorsal **B** lateral views of head and thorax of imago **C** dorsal **D** frontal views of anterior soldier head capsule (white arrows mark frontal flange, yellow arrows mark frontolateral ridge, and black arrows mark protuberance of frontolateral ridge).

***Imago*** (Fig. 2A, B). Single female damaged: distal half of left wings torn, right wings absent. Head capsule black; pronotum brownish orange. Compound eye small, nearly circular. Ocellus very small, circular, black; difficult to see as there is no contrast with head capsule cuticle; ocellus well removed from eye margin. Head vertex and frons not depressed; frons with faint rugosity; covered with dozens of erect setae ca 0.15-mm-long. Pronotum slightly wider than head capsule; anterior margin slightly incised; posterolateral corners evenly rounded, posterior margin narrowly concave. Pronotum pilosity congruent with vertex. Antennae with at least 16 articles, basal article relative lengths 1>2=3>4. Forewing anterior half as per genus; median vein very short, joins radial 0.9 mm from wing scale. Wing scale covered with about 20 setae of similar length and density as those on head and pronotum. Wing membrane smokey brown, covered with darker nodules. Legs dark brown. Arolium present. Measurements (maximum, mm): head width 1.31, pronotum width 1.43, eye diameter 0.32, ocellus diameter 0.01, and body length 7.9.

**Figure 3.**
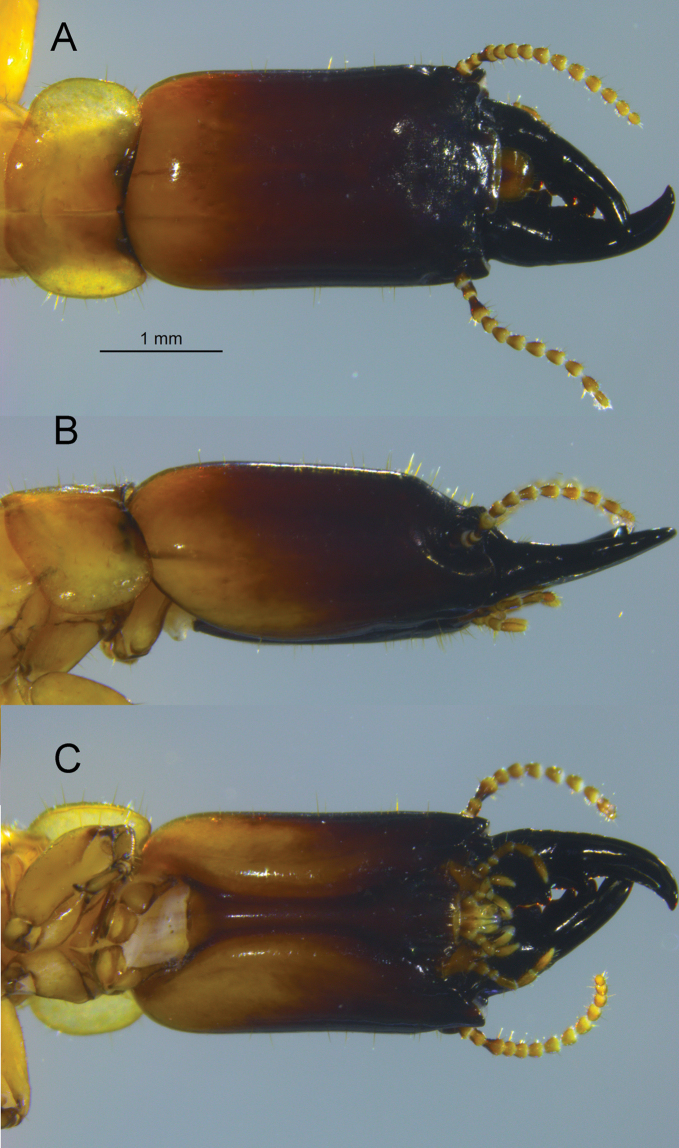
*Rugitermes tinto* sp. nov. soldier head and pronotum. **A** Dorsal **B** lateral **C** ventral view.

***Soldier*** (Figs 2C, D, 3). Head capsule, in dorsal view, dark castaneous brown from posterior margin of postclypeus to posterior one fourth; posterior one fourth grading from dark reddish brown to brownish orange at occiput. In ventral view, head capsule coloration grades gradually from dark castaneous brown to brownish orange with exception of postmentum which remains dark reddish brown at posterior. Pronotum yellowish with reddish interior, in obvious contrast with head capsule coloration. Head capsule long, rectangular; lateral margins very slightly concave in middle, covered with a few setae except at frons where setae are denser. Pronotum much wider than long; with scattered setae, denser along lateral margins; anterior margin weakly incised. In dorsal view, the frontal flange forms a weak hemispherical border surrounding the frons. The frontal flange forms a 30° angle with plane of vertex. Frons finely rugose. In dorsal view (Fig. 2C), frontolateral ridges form shelves on each side of the frons almost in-line with posterior margin of postclypeus. The outer margins of each ridge are adorned with protuberance forming acute angles of ca 65°. In frontal view, the ridges rise slightly at their protuberances; dorsal margins of antennal carinae (“sockets”) positioned well below ridges. Eye spots small, concolorous with head capsule. Third antennal article club-shaped, about twice as long as second and fourth articles. Mandibles about half length of head capsule; outside margin of each blade curving gradually, with very slight hump at base. Measurements in mm [mean (range, *N* = 6)]: Head length lateral base of mandibles: 2.83 (2.47–2.99), max. head width 1.72 (1.52–1.90), max. head height with postmentum 1.42 (1.29–1.47), max. pronotum width 1.86 (1.58–2.04), max. pronotum length 0.90 (0.86–0.95), third antennal article length 0.19 (0.18–0.20).

#### Type material examined.

***Holotype*** (soldier). Colombia: Risaralda, Pereira (4.810, -75.695); 1410 meters a.s.l., 11APR1998, J. Navarro, A. Arevalo; two soldiers (one labelled holotype), one damaged female imago University of Florida Termite Collection (UFTC) no. CO919, subsample from Colección Entomológica Forestal Universidad Distrital “Francisco José de Caldas” (CEFUDFJC) no. 009942 of which remains one soldier and three pseudergates.

#### Other material examined.

Colombia: Cundinamarca, Villeta (5.017, -74.467); 842 meters a.s.l., no date, A. Moreno; one soldier and two pseudergates; CEFUDFJC no. 009940. Colombia: Risaralda, Pereira (4.810, -75.695); 1410 meters a.s.l., 11APR1998, J. Navarro, A. Arevalo. Same location; two soldiers, three pseudergates; CEFUDFJC no. 009942.

#### Distribution.

*Rugitermes tinto* is known from the Cauca River Valley montane ecoregion (Pereira) which has a mesic climate (Olson et al. 2001) and from the higher slopes of the Magdalena River Valley ecoregion (Villeta) which is characterized by more xeric forests (Sánchez-Cuervo et al. 2012).

#### Etymology.

“Tinto” is the Colombian name for plain black coffee which is reminiscent of the dark coloration of the *R. tinto* soldier head capsule. The type locality of *R. tinto*, Pereira, is also in the major coffee growing area of Colombia.

**Figure 4.**
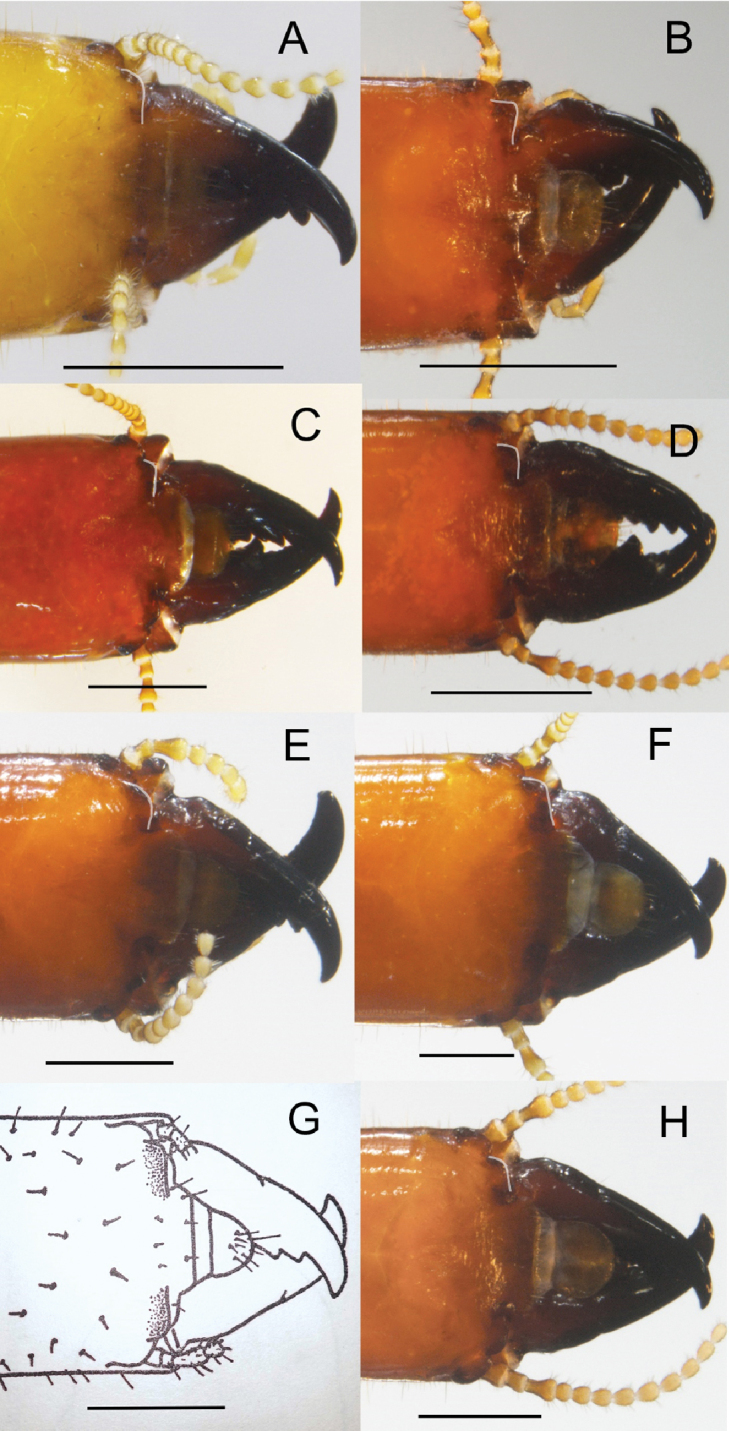
Anteriodorsal views of *Rugitermes* soldier heads from South America. Margins of left frontolateral ridges enhanced. **A***R. bicolor*, PU946 **B***R. flavicinctus*, TT88 **C***R. laticollis*, EC1465 **D***R. magninotus*, PU1087 **E***R. occidentalis*, AG380 **F***R. niger*, AG500 **G***R. nodulosus* (modified from fig. 14, Krishna 1961) **H***R. rugosus* PA1186. Accession numbers from UFTC (Scheffrahn 2019b). Scale bars: 1 mm.

## Discussion

Authoritative records of the kalotermitid diversity for Colombia are reported almost entirely from the Caribbean Region (Casalla et al. 2016a, b; Snyder 1925; Krishna and Emerson 1962; Scheffrahn 2019a, b). An Andean termite survey by Parra and Soto (1992) is a remarkable exception as they report ten kalotermitids from this region. Except for *Cryptotermes brevis* (Walker), their generic and specific assignments are mostly incorrect. Furthermore, the drawings by Parra and Soto (1992) do not allow for positive identification below family. Aside from *R. tinto* and *C. brevis*, the only other confirmed kalotermitid from Andean Colombia is *Glyptotermes truncatus* (Krishna & Emerson, 1962).

It is hoped that future termite collecting in Andean Colombia will uncover many new termite species and expand known species distribution records. The Magdalena River Valley recently also yielded a new non-kalomitid termite, *Rhynchotermes armatus*Scheffrahn (2019c).

## Supplementary Material

XML Treatment for Rugitermes tinto
